# Evaluation of serum adiponectin as a marker of insulin resistance in women with polycystic ovarian syndrome: a comparative cross-sectional study

**DOI:** 10.1186/s12958-024-01196-9

**Published:** 2024-02-20

**Authors:** Olugbenga Ojatokunbo Runsewe, Abiodun Adeniyi Adewunmi, Gbenga Olorunfemi, Abimbola Tawaqualit Ottun, Ayokunle Moses Olumodeji, Babalola Ogungbemile, Tamramat Iyabo Runsewe-Abiodun

**Affiliations:** 1 Department of Obstetrics and Gynaecology, Lagos Island Maternity Hospital, Lagos, Nigeria; 2 Department of Obstetrics and Gynaecology, Lagos State University College of Medicine, Lagos State University, Lagos, Nigeria; 3 Department of Obstetrics and Gynaecology, Lagos State University Teaching Hospital, Lagos, Nigeria; 4 Division of Epidemiology and Biostatistics, University of Witwatersrand, Johannesburg, South Africa; 5 Department of Paediatrics, Olabisi Onabanjo University Teaching Hospital, Sagamu, Nigeria

**Keywords:** PCOS, Insulin resistance, Adiponectin

## Abstract

**Background:**

Insulin resistance (IR) is known to be prevalent amongst women with polycystic ovarian syndrome (PCOS). Its presence has been linked to chronic anovulation and marked long term complications in women. Hence, identification and treatment of IR in women with PCOS is required to prevent the metabolic and reproductive complications of the disease. The aim of this study is to determine if serum adiponectin could be used as a surrogate marker for insulin resistance among women with PCOS.

**Materials and methods:**

A total number of 148 consenting women with PCOS diagnosed using the Rotterdam criteria were recruited for this study. Fifty-two of these women had insulin resistance were compared with 96 of the women who did not have insulin resistance. The serum Adiponectin levels, fasting blood glucose and fasting insulin levels were assayed in all study participants. Insulin resistance was assessed in all the study participants using the Homeostasis Model Assessment for Insulin Resistance (HOMA-IR). Data were analyzed using relevant inferential statistics at 95% confidence interval and *p* value of < 0.05.

**Results:**

The prevalence of insulin resistance among the study participants was 35.1%. Majority of the women (83.1%) had a high body mass index (BMI). More than half (68.2%) of the participants were in the age range of 21-30years and 76.4% (113) were nulliparous. There was no statistically significant difference in the median adiponectin level among insulin resistant (3.735 ug/ml) and non-insulin resistant participants vs. (3.705 ug/ml) (*p* = 0.6762). Both univariate and multivariate regression analysis did not show a statistically significant relationship between adiponectin and insulin resistance in PCOS.

**Conclusion:**

The prevalence of insulin resistance in women with PCOS is high and serum adiponectin is not a suitable surrogate marker of insulin resistance in women with PCOS.

## Introduction

Polycystic ovarian syndrome (PCOS) is a condition characterized by menstrual dysfunction and clinical features of hyperandrogenism [1]. PCOS is a highly prevalent endocrine and metabolic disorder affecting women in the reproductive age group [2]. The major cause of this syndrome remains largely unknown, but findings from several studies suggests that PCOS might be a complex multigenic disorder with strong epigenetic and environmental influences, including diet and lifestyle factors [3]. Polycystic ovarian syndrome is commonly associated with insulin resistance, obesity, metabolic disorders and cardiovascular risk factors [3].

Polycystic ovarian syndrome is a major challenge to public health due to its metabolic, reproductive and psychological features [4]. PCOS has a prevalence of 5–10% in women of reproductive age with variance among race and ethnicities [5]. The highest reported prevalence has been 52% among the South Asian immigrants of whom 49.1% had menstrual irregularity [6]. A previous study done in Southern Nigeria reported a prevalence of 12.2% [6].

PCOS is commonly diagnosed using Rotterdam criteria and is based on two out of three features: oligo- or an-ovulation, hyperandrogenism (clinical or biochemical), and polycystic ovaries [7]. Insulin resistance and hyperinsulinemia plays an important role in the pathogenesis of this condition [7]. Insulin resistance is prevalent among women with PCOS and this is independent of obesity or body mass index (BMI) [8]. It is believed that about 50–70% of women with PCOS would have Insulin resistance [8]. Insulin and hyperinsulinemia have been implicated in the pathogenesis as well as the immediate and long-term complications of PCOS [9].

Insulin resistance have been found to be involved in the development of metabolic syndrome and cardiovascular diseases in women with PCOS [10]. Hence, the determination of insulin resistance and its management is particularly important for women with PCOS. However, measuring insulin resistance is neither simple nor necessarily accurate [10]. Several methods for determining insulin resistance such as the hyperinsulinemic euglycemic clamp which is the gold standard and others like the homeostatic model assessment of insulin resistance (HOMA-IR) are cumbersome, invasive and time consuming [11]. Thus, several surrogate markers have been proposed to facilitate and improve the determination of insulin resistance. One of such markers is Adiponectin, which is an adipocytokine secreted by mature adipocytes found in low levels in women with insulin resistance [10].

There are suggestions that adiponectin levels could be related to insulin resistance in PCOS [12]. However, the relationship between adiponectin levels and insulin resistance in PCOS continues to be disputed. Various studies present conflicting findings; some indicate lower adiponectin levels in PCOS, regardless of Body Mass Index (BMI) [13–15], while others report comparable adiponectin levels in BMI-matched individuals with PCOS and controls [16, 17]. Results of some other studies support the finding that adiponectin levels are associated with insulin resistance [12, 18]. Despite the growing body of evidence in diverse populations, there is a notable paucity of data on this matter in sub-Saharan African populations. Hence, this study evaluated if serum adiponectin could be used as a surrogate marker for insulin resistance. The specificity of the study population, comprising African women, addresses a gap in the existing literature, which often lacks representation from sub-Saharan African populations.

## Materials and methods

### Study design

This was a comparative cross-sectional study that involved comparing 52 women diagnosed with PCOS and insulin resistance to 96 women diagnosed with PCOS without insulin resistance. All participants provided written informed consent and completed a semi-structured questionnaire. Blood samples were collected and analyzed for levels of fasting blood glucose, fasting insulin, and serum adiponectin.

### Study area and population

The study took place at Lagos State University Teaching Hospital Ikeja and Lagos Island Maternity Hospital, Lagos. It included women of reproductive age who had been diagnosed with PCOS according to the Rotterdam criteria in the Gynecology Clinics at the aforementioned hospitals.

### Sampling

Convenience sampling was utilized to recruit consenting women with PCOS who attended clinic days at both hospitals consecutively until the desired sample size was achieved. The study spanned a period of 2 years (January 21, 2020, to January 20, 2022). Participants were categorized into two groups: those with PCOS and insulin resistance as cases, and those with PCOS without insulin resistance as controls.

### Sample size estimation

The sample size formula for comparative study was used in calculating the sample size [19]. The formula being as follows:

$${{\rm{Sample}}\,{\rm{size}}{\mkern 1mu} {\rm{ = }}{\mkern 1mu} \frac{{{\rm{r + }}{\mkern 1mu} {\rm{1/r}}\left( {{{\rm{P}}^{\rm{x}}}} \right){\mkern 1mu} \left( {{\rm{1 - }}{{\rm{P}}^{\rm{x}}}} \right){{({\rm{Z}}\beta {\mkern 1mu} {\rm{ + }}{\mkern 1mu} {\rm{Z}}\alpha )}^{\rm{2}}}}}{{{{\left( {{{\rm{P}}_{\rm{1}}}{\rm{ - }}{\mkern 1mu} {{\rm{P}}_{\rm{2}}}} \right)}^{\rm{2}}}}}}$$

r – Ratio of control to cases, this was taken as 1.

*P*^x^ – the average proportion of PCOS with IR (exposed cases) + proportion of control (PCOS without IR)/ 2.

Zβ– Standard normal variant for power which is 1.28 and 0.8 for 90% and 80% power respectively (1.28 for 90% power will be used).

Zα– Standard normal variant for level of significance = 1.96.

*P*_1_ – *P*_2_ = Effect size of difference in proportion expected based on previous studies. P_1_ is the expected proportion in study group based on maximum available proportion from previous studies while P_2_ is the proportion in control based on maximum available proportion from previous studies [20].

$$\begin{array}{l}{\rm{P1 = }}\,{\rm{0}}{\rm{.36}}\,\left( {{\rm{20}}} \right)\\{\rm{P2 = }}\,{\rm{0}}{\rm{.64}}\,\left( {{\rm{20}}} \right)\\{\rm{Sample}}\,{\rm{size}}\,{\rm{ = }}\frac{{{\rm{1 + 1/1}}\left( {{\rm{0}}{\rm{.36 + }}\,{\rm{0}}{\rm{.64/2}}} \right)\left( {{\rm{1 - }}\left( {{\rm{0}}{\rm{.36 + 0}}{\rm{.64/2}}} \right){\rm{ }}} \right){{\left( {{\rm{1}}{\rm{.28 + 1}}\,{\rm{.96}}} \right)}^{\rm{2}}}}}{{{{\left( {{\rm{0}}{\rm{.36 - 0}}{\rm{.64}}} \right)}^{\rm{2}}}}}\\{\rm{ = }}\frac{{{\rm{2}}\left( {{\rm{0}}{\rm{.50}}} \right)\left( {{\rm{0}}{\rm{.50}}} \right)\left( {{\rm{10}}{\rm{.498}}} \right)}}{{{\rm{0}}{\rm{.0784}}}}\\{\rm{ = }}\,\frac{{{\rm{2}}\,\left( {{\rm{0}}{\rm{.25}}\,{\rm{X}}\,{\rm{10}}{\rm{.498}}} \right)}}{{{{\left( {{\rm{0}}{\rm{.0289}}} \right)}^{\rm{2}}}}}\\{\rm{ = }}\,\frac{{{\rm{2}}\,{\rm{X}}\,{\rm{2}}{\rm{.6245}}}}{{{\rm{0}}{\rm{.0784}}}}\,\,\,\,\,\,{\rm{ = 67}}\end{array}$$

With the attrition rate of 10%, the minimum sample size for this study was 74 (67 + 7) participants per group as 10% [7] of the samples size was added to the ‘calculated sample size’ to account for the non-response rate. So, the total sample size for this study was calculated to be 148.

### Data collection

Participation in the study was contingent upon obtaining written consent from women. Recruitment was conducted consecutively until the predetermined sample size was reached. Following eligibility confirmation, participants were situated in a quiet environment ensuring individual privacy. A semi-structured questionnaire was utilized to gather baseline demographic data and reproductive characteristics. For participants with no education, the questionnaire was administered by the researcher, while others self-administered it.

### Specimen collection

At the second gynaecology clinic visit where participants came for in a fasting state, blood samples were taken. Ten millilitres of blood were collected aseptically from the antecubital fossa vein with minimal stasis using pyrogen free disposable needles and syringes. Five millilitres of the blood were put into two separate labelled specimen tubes each: a Serum Separator Gel tube for the Serum Adiponectin and Serum Fasting Insulin assay and a Fluoride Oxalate tube for the Fasting Plasma Glucose assay. These specimen tubes were transported to the laboratory within 1 to 2 h of specimen collection where they were stored at -20^0^C till analysed within 72 h.

### Specimen analysis

#### Serum adiponectin

Serum Adiponectin was assayed using a solid phase enzyme-linked immunosorbent assay. The assay was done using Abcam’s Human Adiponectin ELISA kit which measures the quantity of adiponectin in serum.

#### Fasting serum insulin

Fasting serum Insulin was assayed using a solid phase enzyme-linked immunosorbent assay. The assay was done using the Accu-Bind ELISA Microwells manufactured by Monobind Inc, USA.

#### Fasting plasma glucose

Fasting plasma glucose was assayed using Glucose Mono Reagent (Glucose Oxidase/Peroxidase method) manufactured by Atlas Medical, United Kingdom.

### Determination of insulin resistance (IR)

Insulin Resistance was determined in all the study participants using the Homeostasis Model Assessment for Insulin Resistance (HOMA-IR) which was calculated as;

$$\begin{array}{l}HOMA - IR = fasting\,insulin\,(\mu U/ml)\, \times \,\\fasting\,glucose\,(mmol/l)/22.5\left( {normalizing\,factor} \right).\end{array}$$

A HOMA-IR value of > 2 was considered as Insulin Resistance for this study [12].

HOMA-IR value of ≤ 2 was considered without Insulin Resistance in this study.

#### Data analysis

The SPSS version 22 was used for data entry, validation and analysis. Frequency tables was generated for all the categorical variables. To test for association between categorical variables in contingency tables, the chi-square test was used with *p*-values of less than 0.05 taken as significant. Multivariate regression analysis was used to evaluate for an association between serum adinopectin levels and insuline resistance in PCOS.

## Results

A total number of 148 participants was recruited in this study. Table 1 shows the comparison of the socio-demographic characteristics of the study population. The mean age of the participants was 26.99 ± 4.88 years. Majority (68.2%) of the participants were within the age range 21–30 years. 58.78% of the participants were married, 67.57% had tertiary education and 76.35% were para 0. Only about 16.89% of the participants had normal body mass index. There was a statistically significant difference in the body mass index between participants with insulin resistance PCOS and those with non-insulin resistance PCOS. Thus, the Body mass index among participants with insulin resistance PCOS was higher than the Body mass index of participants with non-insulin resistant PCOS (31.07 ± 5.05 Vs 28.82 ± 6.37, *p*-value = 0.006) and this was statistically significant. However, there was no statistically significant difference in the other socio-biological characteristics among participants with insulin resistance as compared to those without insulin resistance PCOS.

**Table 1:** Comparison of the socio-demographic characteristics of the study population

Variables	(PCOS + IR) *n* = 52	PCOS - IR *N* = 96	Total *N* = 148 (%)	*P*-value
**Age Distribution (Years)**				
Mean (± SD)	27.10 ± 5.05	26.77 ± 4.59	26.99 ± 4.88	0.69^#^
< 20	5 (9.62)	7 (7.29)	12 (8.11)	0.86^
21–30	35(67.31)	66 (68.75)	101(68.24)	
31–40	12 (23.08)	22(22.92)	34 (22.97)	
41–49	0(0.00)	1(1.04)	1 (0.68)	
**Marital status**				
Married	33 (63.46)	54 (56.25)	87(58.78)	0.39^$^
Single	19 (36.54)	42 (43.75)	61(41.22)	
**Educational Level**				
None	1 (1.92)	0 (0.00)	1(0.68)	0.12^
Primary	2 (3.85)	4 (4.17)	6(4.05)	
Secondary	19(36.54)	22 (22.92)	41(27.70)	
Tertiary	30 (57.69)	70 (72.92)	100 (67.57)	
**Parity**				
0	37 (71.15)	76 (79.17)	113(76.35)	0.27^$^
≥ 1	15 (28.85)	20 (20.83)	35(23.65)	
**Miscarriages/abortion**				
0	34 (65.38)	53 (55.79)	87 (59.18)	0.48^
1	9 (17.31)	22 (23.16)	31 (21.09)	
2	8 (15.38)	19 (20.00)	27 (18.37)	
3	0 (0.00)	1 (1.05)	1 (0.68)	
5	1 (1.92)	0 (0.00)	1 (0.68)	
**BMI (Kg/m** ^**2**^ **)**	31.07 ± 5.05	28.82 ± 6.37	29.84 ± 6.08	0.006^#^
Underweight < 18.5	0 (0.00)	5 (5.21)	5 (3.38)	0.003^
Normal (18.5–24.9)	2 (3.85)	23 (23.96)	25 (16.89)	
Overweight (25.0-29.9)	23 (44.23)	28 (29.17)	51 (34.46)	
Obesity I (30.0-34.9)	16(30.77)	25 (26.04)	41(27.70)	
Obesity II (35.0-39.9)	7(13.46)	13 (13.54)	20 (13.51)	
Extreme Obesity (40.0+)	4 (7.69)	2 (2.08)	6 (4.05)	

^#^ t-test; ^Fischer’s exact test; ^$^ Pearson’s Chi-square, PCOS + IR = Women with Polycystic Ovarian Syndrome and Insulin Resistance, PCOS - IR = Polycystic Ovarian Syndrome without Insulin Resistance

Table 2 shows that there was no statistically significant relationship between serum adiponectin and the prevalence of insulin resistant PCOS in the study.

**Table 2:** Comparison of serum Adiponectin levels, fasting blood glucose and fasting insulin levels among the study population

Serum Adiponectin levels (ug/ml)	PCOS + IR *n* = 52 (%)	PCOS - IR *n* = 96 (%)	*P*-value
**Median, IQR**	3.74 (1.06–23.76)	3.71(1.48–20.98)	0.68^#^
**Low (< 3.5)**	25 (48.08)	46 (47.92)	0.81^$^
**Normal (3.5–22.4)**	13 (25.00)	28 (29.17)	
**High (> 22.4)**	14 (26.92)	22(22.92)	
**Fasting Blood glucose levels (mmol/L)**			
Median, IQR	5.03(3.85–5.75)	4.55 (3.8–5.22)	0.029^#^
< 5.6	35 (67.31)	86 (89.58)	0.003
5.6–6.9	12 (23.08)	7 (7.29)	
≥ 7	5(9.62)	3 (3.13)	
**Fasting Insulin Levels (µU/ml)**			
Median, IQR	14.64(10.89–19.98)	4.22 (2.62–7.56)	< 0.001
< 0.7	0(0.00)	0(0.00)	< 0.001
0.7-9.0	3 (5.77)	84 (87.50)	
> 9.0	49 (94.23)	12 (12.50)	

^#^: Mann-Whitney U test^; $^ Pearson’s Chi-square

PCOS + IR = Women with Polycystic Ovarian Syndrome and Insulin Resistance, PCOS - IR = Polycystic Ovarian Syndrome without Insulin Resistance

From Table 2, there was a statistically significant relationship between fasting blood sugar, fasting insulin level and insulin resistant PCOS. Thus, the proportion of women with impaired or high sugar level among the insulin resistant PCOS group was higher than the proportion of women with impaired or high sugar level among women with non-resistant PCOS.

Similarly, the insulin level among participants with Insulin resistant PCOS was higher than the insulin level among the participants with non-insulin resistant PCOS.

**Fig. 1:**
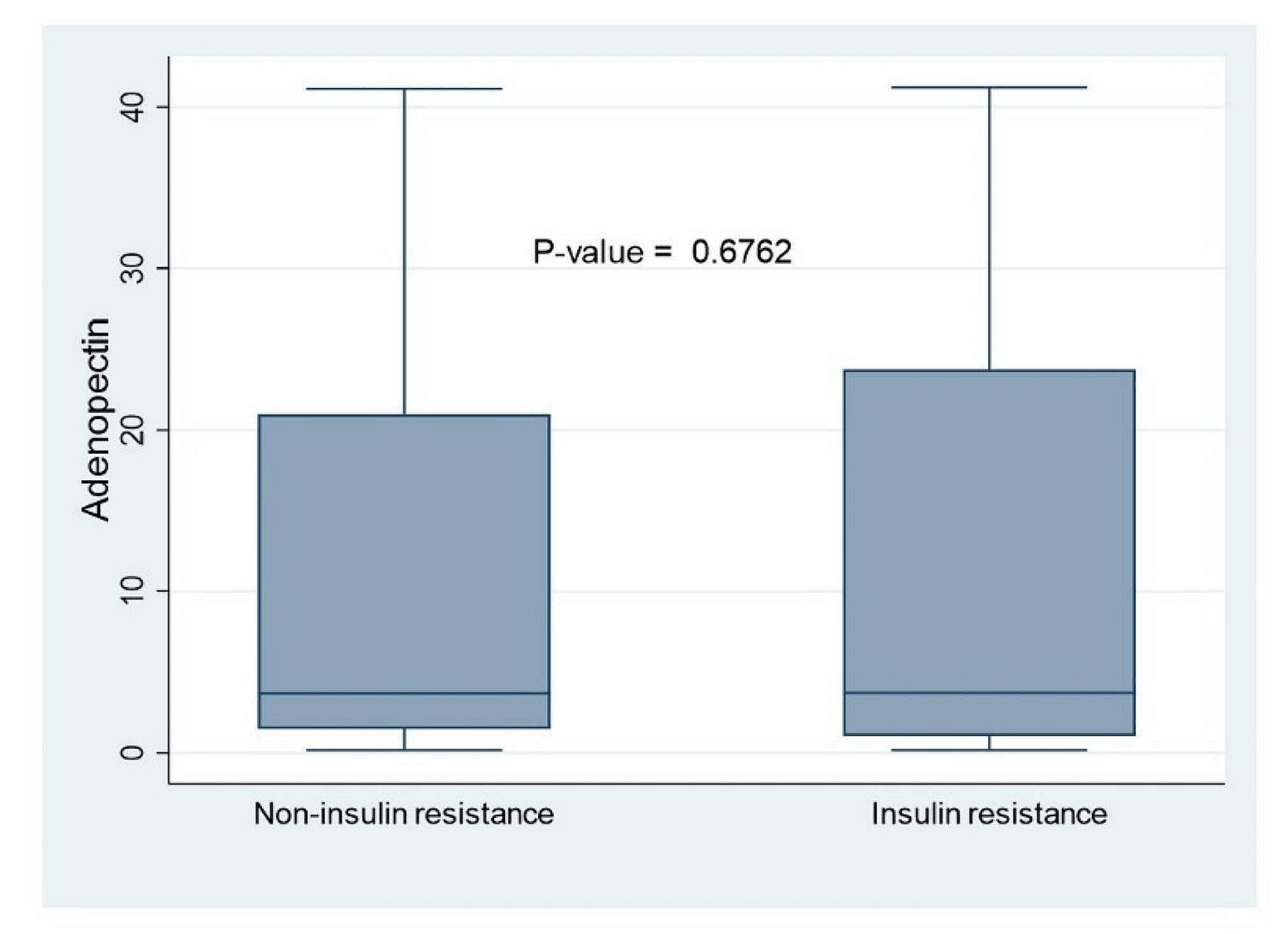
Box plot of the distribution of Adiponectin levels among insulin and non-insulin resistant participants

There was no statistically significant difference in the median Adiponectin levels among insulin resistant and non-insulin resistant participants. 3.735ug/ml (1.055–23.7585) Vs 3.705ug/ml (1.48–20.981), *p*-value = 0.68) as seen in Fig. 1. The maximum and minimum adiponectin level among insulin resistant and non-insulin resistant participants was 0.18ug/ml & 41.23 ug/ml and 0.18ug/ml & 41.128 ug/ml respectively.

Table 3 shows that there was no statistically significant relationship between age, fasting blood sugar, fasting insulin level and adiponectin level among insulin resistant and non-insulin resistant PCOS participants. There is a significant association between BMI and adiponectin levels in PCOS women without insulin resistance, with a *p*-value of 0.03. This is not observed in PCOS women with insulin resistance.

**Table 3:** Serum adiponectin levels in relation to some variables

Variables	PCOS + IR *n* = 52 (%)			*P*-value	PCOS – IR *n* = 96 (%)			*P*-value
	SERUM ADIPONECTIN LEVEL (ug/ml)				SERUM ADIPONECTIN LEVEL (ug/ml)			
	LOW < 3.5	NORMAL 3.5–22.4	HIGH > 22.4		LOW < 3.5	NORMAL 3.5–22.4	HIGH > 22.4	
**BMI (Kg/m** ^**2**^ **)**								
Underweight < 18.5	0 (0.00)	0 (0.00)	0 (0.00)	0.62	4 (8.70)	1 (3.57)	0 (0.00)	0.03
Normal (18.5–24.9)	2 (8.00)	0 (0.00)	0 (0.00)		14 (30.43)	8 (28.57)	1 (4.55)	
Overweight (25.0-29.9)	13 (52.00)	6 (46.15)	4 (28.57)		15 (32.61)	9 (32.14)	4 (18.18)	
Obesity I (30.0-34.9)	6 (24.00)	5 (38.46)	5 (35.71)		9 (19.57)	5 (17.86)	11(50.00)	
Obesity II (35.0-39.9)	2 (8.00)	1 (7.69)	4 (28.57)		4 (8.70)	4 (14.29)	5 (22.73)	
Extreme Obesity (40.0+)	2 (8.00)	1 (7.69)	1 (7.14)		0 (0.00)	1 (3.57)	1 (4.55)	
**Age (Years)**								
≤ 20	2 (8.00)	1 (7.69)	2 (14.29)	0.92	3 (6.52)	2 (7.14)	2 (9.09)	0.96
21–30	18 (72.00)	9 (69.23)	8 (57.14)		31 (67.39)	21 (75.00)	14 (63.64)	
31–40	5 (72.00)	3 (69.23)	4 (57.14)		11 (23.91)	5 (17.86)	6 (27.27)	
41–49	0 (20.00)	0 (23.08)	0 (28.57)		1 (2.17)	0 (0.00)	0 (0.00)	
**Fasting Sugar level**								
LOW (< 5.6)	20 (80.00)	5 (38.46)	10 (71.43)	0.11	42 (91.30)	26 (92.86)	18 (81.82)	0.61
NORMAL (5.6–6.9)	4 (16.00)	5 (38.46)	3 (21.43)		3 (6.52)	1 (3.57)	3 (13.64)	
HIGH (≥ 7)	1 (4.00)	3 (23.08)	1 (7.14)		1 (2.17)	1 (3.57)	1 (4.55)	
**Fasting Insulin level**								
< 0.7	0 (0.00)	0 (0.00)	0 (0.00)	0.22	0 (0.00)	0 (0.00)	0 (0.00)	0.22
0.7-9.0	1 (4.00)	2 (15.38)	0 (0.00)		38 (82.61)	27 (96.43)	19 (86.36)	
> 9.0	24 (96.00)	11 (84.62)	14 (100.00		8 (17.39)	1 (3.57)	3 (13.64)	

PCOS + IR = Women with Polycystic Ovarian Syndrome and Insulin Resistance, PCOS - IR = Women with Polycystic Ovarian Syndrome without Insulin Resistance

Majority of the participants (89.19%) had menstrual irregularity, while just above half had infertility (54.73%), 56.08% had hirsutism, and 52.70% of the participants had acne. Only few participants (8.11%) had alopecia. There was no statistically significant relationship between the symptoms and insulin resistant PCOS. There was no statistically significant relationship between duration of symptoms and insulin resistant PCOS. (Table 4)

**Table 4:** Comparison of presenting symptoms and duration of symptoms

PRESENTING SYMPTOMS	PCOS + IR *n* = 52	PCOS – IR *n* = 96	Total	*P*-value
INFERTILITY				
Yes	29 (55.77)	52 (54.17)	81 (54.73)	0.85
No	23 (44.23)	44 (45.83)	67 (45.27)	
**MENSTRUAL IRREGULARITIES**				
Yes	49 (94.23)	83 (86.46)	132 (89.19)	0.18
No	3 (5.77)	13 (13.54)	16 (10.81)	
**HIRSUTISM**				
Yes	30 (57.69)	53 (55.21)	83 (56.08)	0.77
No	22 (42.31	43 (44.79)	65 (43.92)	
**ACNE**				
Yes	28 (53.85)	50 (52.08)	78 (52.70)	0.84
No	24 (46.15)	46 (47.92)	70 (47.30)	
**ALOPECIA**				
Yes	5 (9.62)	7 (7.29)	12 (8.11)	0.62
No	47 (90.38)	89 (92.71)	136 (91.89)	
**DURATION OF SYMPTOMS (YEARS)**				
< 1	4 (7.69)	11 (11.46)		0.83
1–5	42 (80.77)	75 (78.13)		
≥ 5	6 (11.54)	9 (9.38)		
No Symptom*	0 (0.00)	1 (1.04)		

PCOS + IR = Women with Polycystic Ovarian Syndrome and Insulin Resistance, PCOS - IR = Women with Polycystic Ovarian Syndrome without Insulin Resistance

* The diagnosis of PCOS was made in this single individual who had presented with an incidental finding of polycystic ovaries on ultrasound and mild biochemical hyperandrogenism noted on further evaluation

Following univariable regression model, BMI and fasting insulin were statistically associated with insulin resistant PCOS. However, there was no statistically significant relationship between adiponectin and insulin resistant PCOS at both univariable and multivariable regression modelling (Table 5). Nonetheless, there was a 2.3fold odds of diagnosing insulin resistant PCOS for every unit increase in the fasting insulin level after correcting for confounding variables. (Adj OR: 2.31, 95%CI: 1.61–3.31, *P*-value < 0.001)

**Table 5:** Univariable and multivariable logistic regression of the predictors of insulin resistant PCOS

Variable	Univariable			Multivariable		
	Unadjusted Odds ratio	95%CI	*P*-value	Adjusted* Odds ratio	95%CI	*P*-value
**Age**	0.99	0.92–1.06	0.69	1.13	0.96–1.32	0.14
**≤ 20**	**1.00**	**Reference**	**Reference**	**1.00**	**Reference**	**Reference**
21–30	0.74	0.22–2.51	0.63	4.81	0.36–63.61	0.23
31–40	0.76	0.20–2.93	0.70	9.61	0.58–159.89	0.12
41–49	1.00	-	-	1.00	-	-
**Adiponectin**						
**< 3.5**	**1.00**	**Reference**	**Reference**	**1.00**	**Reference**	**Reference**
3.5–22.4	0.85	0.38–1.94	0.71	1.05	0.21–5.16	0.945
> 22.4	1.17	0.51–2.68	0.71	0.72	0.14–3.73	0.70
BMI	1.08	1.02–1.15	0.007*	1.005	0.88–1.15	0.95
Fasting Insulin	2.17	1.60–2.93	< 0.001*	2.31	1.61–3.31	< 0.001*

*Confounding factors adjusted for were Age and BMI

## Discussion

This study assessed serum adiponectin levels in women with PCOS and compared serum adiponectin in these women with and without insulin resistance. The prevalence of IR among women with PCOS was 35.1% in this study. This is considerably higher than what was reported in a study in Thailand in which 20% of women with PCOS had IR [21]. This may be due to the increased prevalence of deranged fasting blood glucose in this study compared to the one in Thailand (5.4% vs. 3.2%) [21]. Findings from this study shows that the median adiponectin levels among insulin resistant and non-insulin resistant participants was 3.735 (1.055–23.7585 µg/mL) and 3.705(1.48–20.981 µg/mL) respectively and this was similar to a study done in Ireland which reported mean adiponectin levels of 3.03ug/ml in women with non-IR PCOS and 2.88ug/ml in those with IR-PCOS after determining IR using HOMA [22]. However, in another similar study conducted in Bulgaria which reported a mean of 8.85 ± 4.6 ug/ml in women with IR-PCOS and 13.62 ± 7.55 ug/ml in women with non-IR PCOS, the median adiponectin levels are found to be significantly higher than the median adiponectin levels reported in our study. This could be attributed to interracial variation in PCOS [23]. Interracial variation in PCOS and adiponectin levels has been suggested in a study which showed that serum adiponectin levels are lower in African Americans as compared to Caucasians [24]. In this study, there was no statistically significant difference in the median Adiponectin level among insulin resistant and non-insulin resistant participants (*p* = 0.68).

Furthermore, this study has shown that adiponectin may not be a good marker for insulin resistance (IR) in PCOS. There was no statistically significant relationship between adiponectin and insulin resistant PCOS at both univariable and multivariable regression modelling. Correspondingly, results from research in Iran showed that after adjusting for the effect of age, BMI, blood glucose and waist circumference, insulin resistance was not associated with adiponectin levels [25]. The two subpopulations that exist in women with PCOS; one with insulin resistance of possible different etiologies and another without insulin resistance. These etiologies include adiposity, insulin receptor mutation, and unknown causes that could not be readily identified. These undefined interactions, further explains why adiponectin may not be a reliable marker of insulin resistance in the general population of women with PCOS.

However, there was 2.3-fold odds of diagnosing insulin resistant PCOS for every unit increase in the fasting insulin level after adjusting for confounding variables. (Adj OR: 2.31, 95%CI: 1.61–3.31, *p*-value < 0.001). This corroborates with results from another study by Koleva et al., [23] who found fasting plasma glucose and fasting insulin to be significantly elevated in women with IR PCOS, compared to women with non-IR PCOS.

The mean age of the participants in this study was 26.99 ± 4.88 years. This is similar to the mean age reported in studies in India (22 ± 5.0 years years) [26] and Iran (24.8 ± 5.6 years) [25]. This is most probably due to PCOS being a disease that is commoner among young women of childbearing age. The mean BMI of participants in this study was 29.84 ± 6.08 Kg/m^2^, and there is not much difference when compared with other studies. In a study of women with PCOS conducted in South Korea, the mean BMI was 25.1 ± 5.5Kg/m^2^ [27]. Also, the mean BMI of women with PCOS was reported to be 28.4 ± 7.01Kg/m^2^ in Egypt [28]. This seems to suggest that PCOS is common among overweight women of childbearing age. Although these BMIs fall into the overweight category, it is difficult to ascribe this to weight gain due to PCOS alone as many other factors (such as diet, lifestyle and, activity) could be responsible for weight gain. However, in this study, comparison between women with IR-PCOS and non-IR PCOS showed that the BMI of participants with IR-PCOS was higher than the BMI of participants with non-IR PCOS (31.07 ± 5.05 Vs 28.82 ± 6.37, *p*-value = 0.006). After regression analysis, BMI was statistically associated with IR-PCOS. This corroborates with findings from another study which showed that the insulin resistant patients were significantly more obese as shown by three different measures of adiposity; higher BMI (*p* < 0.0001), percentage of body fat (*p* < 0.002) and Waist-to-hip ratio (W/H) (*p* < 0.005) [29]. This highlights the link between IR and obesity/adiposity as measured by different methods.

Evidence from this work shows that majority of the participants had menstrual irregularity (89.19%) and just over half had some noticeable features of hyperandrogenism; hirsutism (56.08%) and acne (52.70%). Likewise in a study in Thailand, 98.4% of women had oligomenorrhea or amenorrhea, and almost half of them (49.2%) had features of hyperandrogenism [21].

## Conclusion

Although this study confirms that the prevalence of insulin resistance amongst women with PCOS is high, it has not been able to prove that serum adiponectin can be used as a surrogate marker for insulin resistance in women with PCOS. Therefore, the determination of insulin resistance in women with PCOS is necessary in view of its high prevalence and implications on women’s health. More local studies are needed to confirm that serum Adiponectin and other adipocytokines can be used as surrogate markers for insulin resistance.

### Strengths

This study contributes to the understanding of metabolic aspects in PCOS patients. Also, its comparative design allows for a direct comparison between women with PCOS with and without insulin resistance, providing insights into the potential differences in serum adiponectin levels associated with insulin resistance.

### Limitation

The study’s findings may be specific to the population in Lagos, and caution should be exercised when generalizing to other regions or populations with different demographic and healthcare characteristics.

## Data Availability

The datasets used and/or analysed during the current study are available from the corresponding author on reasonable request.
